# Distinct increased outliers among 136 rectal cancer patients assessed by γH2AX

**DOI:** 10.1186/s13014-015-0344-5

**Published:** 2015-02-11

**Authors:** Jana Kroeber, Barbara Wenger, Manuela Schwegler, Christoph Daniel, Manfred Schmidt, Cholpon S Djuzenova, Bülent Polat, Michael Flentje, Rainer Fietkau, Luitpold V Distel

**Affiliations:** Department of Radiation Oncology, Friedrich-Alexander-University of Erlangen-Nürnberg, Universitätsstraße 27, D-91054 Erlangen, Germany; Department of Nephropathology, University Hospitals and Friedrich-Alexander-University Erlangen-Nürnberg Institute of Pathology, 91054 Erlangen, Germany; Department of Radiation Oncology, University of Würzburg, 97080 Würzburg, Germany

**Keywords:** γH2AX, DNA double strand breaks, Rectal cancer, Radiotherapy, Individual radiosensitivity

## Abstract

**Background:**

In recent years attention has focused on γH2AX as a very sensitive double strand break indicator. It has been suggested that γH2AX might be able to predict individual radiosensitivity. Our aim was to study the induction and repair of DNA double strand breaks labelled by γH2AX in a large cohort.

**Methods:**

In a prospective study lymphocytes of 136 rectal cancer (RC) patients and 59 healthy individuals were ex vivo irradiated (IR) and initial DNA damage was compared to remaining DNA damage after 2 Gy and 24 hours repair time and preexisting DNA damage in unirradiated lymphocytes. Lymphocytes were immunostained with anti-γH2AX antibodies and microscopic images with an extended depth of field were acquired. γH2AX foci counting was performed using a semi-automatic image analysis software.

**Results:**

Distinct increased values of preexisting and remaining γH2AX foci in the group of RC patients were found compared to the healthy individuals. Additionally there are clear differences within the groups and there are outliers in about 12% of the RC patients after ex vivo IR.

**Conclusions:**

The γH2AX assay has the capability to identify a group of outliers which are most probably patients with increased radiosensitivity having the highest risk of suffering radiotherapy-related late sequelae.

## Background

In radiation therapy there is a need to find a monitoring assay which can predict a patients’ individual radiosensitivity and the risk to develop early and late tissue reaction and to adjust the radiation dose when indicated. A pretreatment identification of cancer patients by a predictive assay of normal-tissue radiosensitivity may allow appropriate adjustment of treatment and could reduce the risk of therapy related side effects. Several different predictive assay strategies were tested, for review see [1].

To monitor the development of DNA double strand breaks (DSB) γH2AX was established as a well-known biomarker and over the last years the γH2AX assay has come into the focus of attention as a predictive assay. H2AX is a member of the histone H2A family [2]. H2AX is rapidly phosphorylated after exposure of cells to ionizing radiation. γH2AX phosphorylation develops in minutes and reaches its maximum after about 30 minutes [3]. The number of DNA DSBs can be directly determined by the number of foci present in the cell shortly after DNA damage [4], since it has been proved that one γH2AX focus represents one DNA DSB [5]. Rothkamm et al. proclaimed that H2AX phosphorylation and γH2AX foci formation are now generally accepted as consistent and quantitative markers of DSBs, applicable even under conditions where only a few DSBs are present [5].

The efficiency of γH2AX detection as a biomarker for DNA DSBs makes this protein a good candidate as a therapeutic marker for improving the efficiency of radiation, drug and other therapies [6-8]. Dickey et al. revealed that γH2AX is a sensitive indicator of DNA DSBs and is therefore a potentially useful tool in the detection of genotoxic stress. Such an indicator could be valuable in monitoring cancer development and progression as well as other instances of cell stress. Future work in this field needs to be directed at moving the γH2AX detection assay to the clinic where it will be used as a practical means to detect cancer and monitor therapeutic progress [9]. Therefore, the aim of this study was to evaluate the importance of the γH2AX assay in the detection of rectal cancer patients with remaining γH2AX foci and a possibly increased radiosensitivity.

## Methods

### Study participants

**Figure 1:**
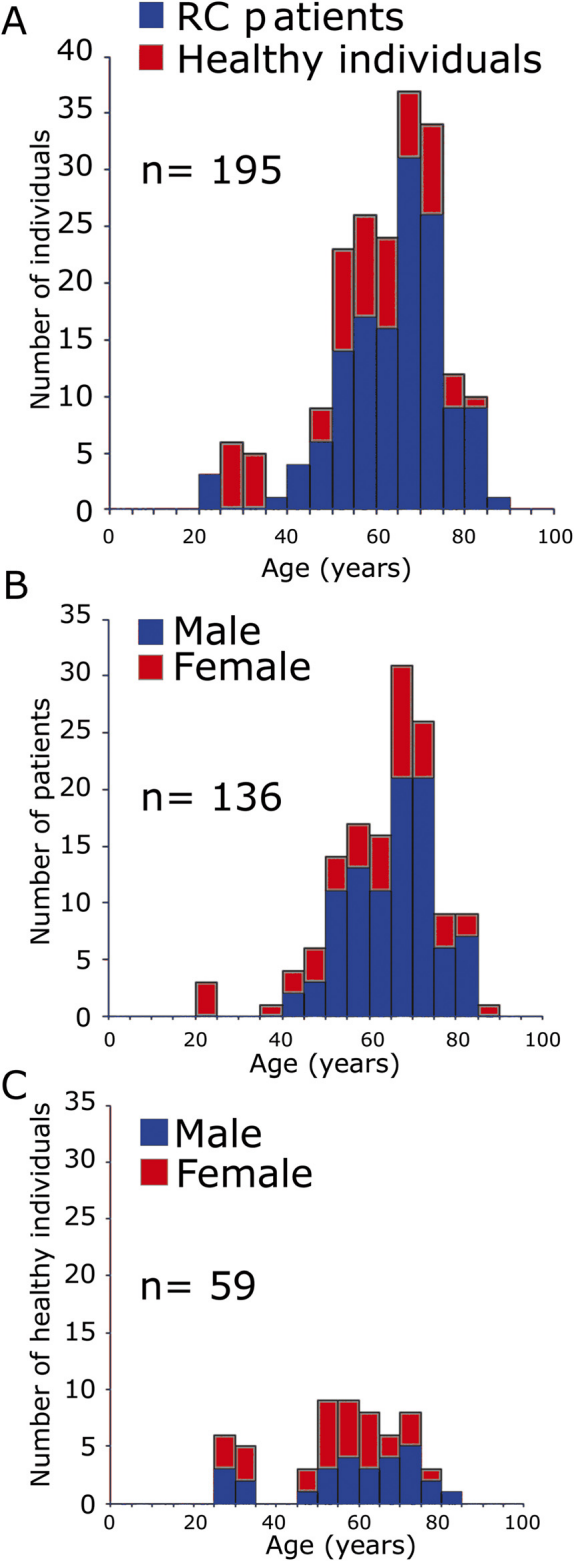
**Distribution by age and gender for the groups of rectal cancer patients (RC) and healthy individuals. (A)** The whole study population separated by patients (blue bars) and healthy individuals (red bars). **(B)** Patients’ group and **(C)** healthy individuals group separated by gender. Female (red bars) and male (blue bars).

The prospective study included a total of 195 individuals. Enrolled were 136 rectal cancer patients (RC) and 59 healthy individuals (Figure 1A). All Patients were treated with neoadjuvant radiochemotherapy and total mesorectal excision surgery. Radiotherapy consisted of 50.4 Gy in 28 daily fractions of 1.8 Gy. The patients’ age was between 23 and 87 years (Figure 1B) with a mean age of 63.7 years. The age of the control group was between 27 and 80 (Figure 1C) with a mean age of 56.0 years. Table 1 shows an overview of stage and radiotherapeutic and chemotherapeutic treatment of all individuals. This study was approved by the ethics review committees of the Friedrich-Alexander-Universität Erlangen-Nürnberg (No. 2725) and all patients and healthy individuals gave their written informed consent. Blood samples were collected shortly before the first irradiation treatment. Afterwards the patients received a conventional fractionation schedule of radiation (1.8 Gy/fraction) up to a total dose of 50.4 Gy.

**Table 1:** **Healthy individuals and rectal cancer (RC) patients’ characteristics**

	**Number of individuals**	**Mean/median age**	**Pathologic stage (UICC) 1/2/3/4 (%)**	**Chemotherapy 5-FU/5-Fu + Oxa/Irinotecan/5-FU + Irinotecan (%)**	**Lymph node metastasis yes/no (%)**	**Total RT dose (Gy)**
Healthy individuals	59	56.0/58.5				
Patients	136	63.7/66.0	0/8.1/75.7/16.2	25.7/64.5/2.9/3.7	89.5/10.5	50.4

### Immunofluorescence Staining

Patients and healthy individuals’ blood samples were split into three samples. One sample was used as control to detect the spontaneous γH2AX foci formation. The second sample was ex vivo irradiated with 0.5 Gy X-rays and 30 minutes incubation time and the third one with 2 Gy and 24 hours incubation time. The two different doses were chosen, because a dose of 2 Gy induces after 30 min such a high number of foci that it is not possible to count the foci accurately. On the contrary, with a dose of 0.5 Gy and 24 hours repair the amount of foci is too low to have sufficient foci numbers. Therefore for the initial γH2AX foci a low and for the remaining γH2AX foci after 24 hours repair time a high dose was chosen. Peripheral blood mononucleated cells (PBMC) were isolated by Ficoll gradient centrifugation and were cytocentrifuged (StatspinCytofuge, Kreatech, Germany) onto a specimen. The samples were fixed with methanol and acetone and afterwards washed in a phosphate-buffered saline with foetal calf serum. The slides were then incubated with a mouse anti-γH2AX antibody (Abcam, Cambridge, UK), washed in PBS and incubated with a secondary goat anti-mouse Alexa 488 fluorescent antibody (Molecular Probes, Karlsruhe, Germany). Afterwards lymphocytes were washed in PBS and mounted with Vectashield mounting medium (Vector Laboratories, Peterborough, UK).

### γH2AX foci recognition

**Figure 2:**
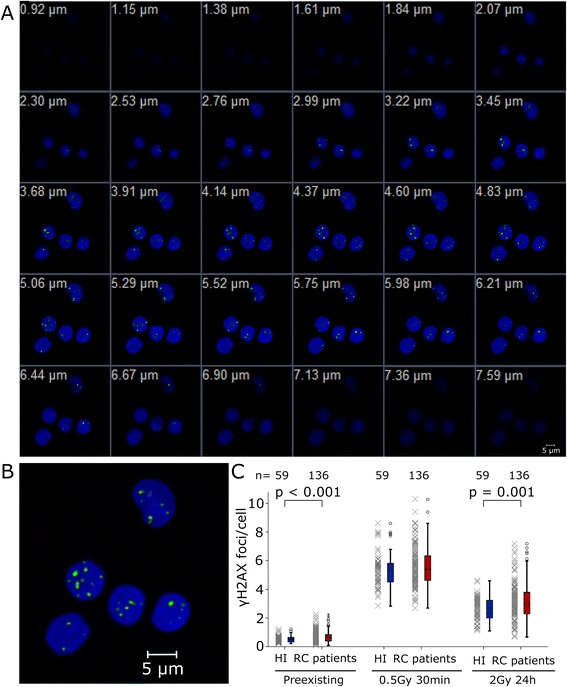
**Immunostained** **γH2AX foci in lymphocytes 24 h post 2Gy IR. (A)** 30 z-sections with a 0.23 μm step size acquired by a laser scanning confocal microscope and **(B)** a resulting combined extended focus image. **(C)** Preexisting, initial and remaining γH2AX foci per cell in 59 healthy individuals (HI, blue) and 136 rectal cancer patients (red).

Fluorescence labelled lymphocytes were visualised by a fluorescence-microscope (Axioplan 2, Zeiss, Göttingen, Germany) and image acquisition software (Metafer 4, MetaSystems, Altlußheim, Germany). Digital images of five optical planes separated by a distance of 0.75 μm were recorded and combined to an extended focus image using the maximum intensity algorithm (Metasystems). An area of 2 mm^2^ (magnification 630×) was captured automatically. For each of the samples at least 1000 lymphocytes were identified using image analysis software (Biomas, Erlangen, Germany). All DAPI-stained nuclei were morphologically considered by eye and apoptotic cells were excluded. Using the image analysis software the γH2AX foci inside each nucleus were counted [10]. The number of mean foci per cell was determined for every individual before irradiation, 30 minutes after 0.5 Gy and 24 h after 2 Gy ionising radiation (IR). A representative image of γH2AX foci location in the lymphocytes was acquired by a laser scanning confocal microscope (LSM710, Zeiss, Göttingen, Germany). 30 z-sections with a 0.23 μm step size were recorded and combined to an extended focus image (Figure 2A, B).

### Statistical analysis

Data analysis and statistics were performed using SPSS 21 for Windows (IBM Corp., Armonk, NY, USA). For comparing the results referred to the different groups, the Kolmogorov–Smirnov test and Lilliefors test were applied for testing normality and data were fitted by a Gaussian distribution. Standard deviations of the Gaussian distributions have been used to designate an individual’s categorization. The cut-off values of 2 and 3 standard deviations are equivalent to the 95% and 99% confidence intervals. Different groups were compared using the two-sample t-test. Graphics were plotted with TechPlot for Windows 3.0.11 (SFTek, Dr. Ralf Dittrich, Braunschweig, Germany).

## Results

γH2AX foci rates in a group of 136 RC patients were compared to 59 healthy individuals (Figure 1). Patients’ characteristics are described in Table 1. Three samples per patient were analysed. Preexisting γH2AX foci were compared to initial foci numbers exposed to 0.5 Gy IR and a 30 minutes waiting period. Remaining γH2AX foci were scored after exposure to 2 Gy IR and 24 hours recovery time.

First it was estimated how many cells had to be counted to obtain reliable γH2AX rates. The suitable minimum number to yield stable γH2AX rates was estimated by a Bland Altman analysis. The agreement between the γH2AX foci rates after every 200 cells was estimated. The value indicating an exact agreement would be 100%. Deviations up- and downward are indicated by higher and lower percentages, respectively. A deviation of ±15% was defined as the range of tolerance. Counting 400 unirradiated lymphocytes, the range for the parameter γH2AX foci rate was very large. When scoring about 600 lymphocytes, 75% of the values were within the defined range of tolerance of z or ±15% of the 1000 lymphocytes values. By scoring 800 lymphocytes 95% of the values were within the defined range of tolerance of ±15%. Though a minimum number of 600 lymphocytes were counted and if it was suitable, about 1000 lymphocytes were scored.

**Figure 3:**
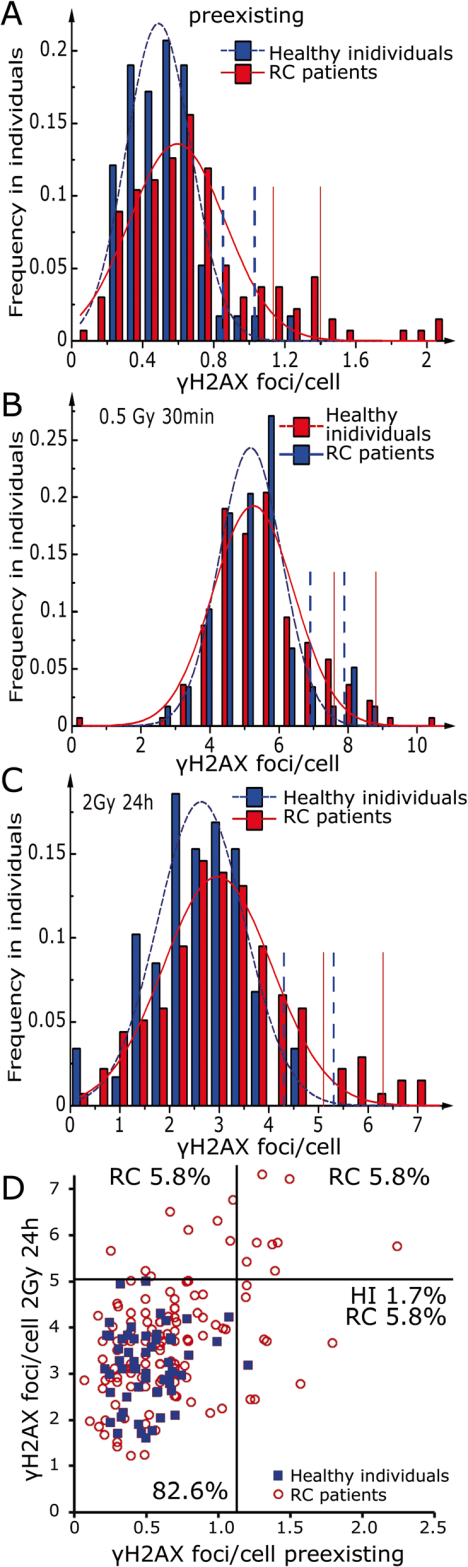
**Frequency distribution of the individual** **γH2AX foci per cell as measured in ex vivo irradiated lymphocytes after immunostaining.** 59 healthy individuals (blue bars) were compared to 136 rectal cancer patients (red bars). The data were fitted using a Gaussian distribution for the **(A)** preexisting γH2AX foci, **(B)** initial γH2AX foci 30 min post IR with 0.5 Gy **(C)** remaining γH2AX foci 24 h post IR with 2 Gy. Dashed blue vertical lines indicate the value of two and three standard deviations from the mean of the healthy individuals and solid red lines the value of two and three standard deviations from the mean of the RC patients, respectively. **(D)** Preexisting γH2AX foci were correlated to remaining γH2AX foci 24 h post IR with 2Gy. HI = healthy individuals, RC = rectal cancer patients.

The preexisting γH2AX foci rates in lymphocytes of RC patients’ were significantly increased compared to the healthy individuals (p < 0.001). No difference between the two groups was found in initial γH2AX rates after 0.5 Gy and 30 minutes incubation time. The residual foci after a dose of 2 Gy and 24 hours recovery time were significantly increased in the RC patients group compared to the healthy individuals (p < 0.001) (Figure 2C). Additionally in the patients group an increased number of individuals with distinct higher γH2AX rates was observed. In order to analyse this effect, γH2AX rates from healthy individuals and patients were classified into suitable divisions of γH2AX foci per cell and a Gaussian fit was performed. This approach was used to evaluate individuals that distinct fell outside the Gaussian distribution and could be identified as outliers (Figure 3). Using the Kolmogorov–Smirnov test, the distributions of the patients and healthy individuals were normally distributed, while the more stringent Lilliefors test showed normality for all but the group of preexisting and remaining γH2AX foci of the healthy individuals. The mean preexisting γH2AX foci of the RC patients’ distribution was increased by 20.4% compared to the control group (Figure 3A). The mean values of the normal distributions for the initial DNA damage were quite similar (Figure 3B), while the mean residual DNA damage of the RC patients was increased by 13% (Figure 3C). In the group of RC patients more outliers were observed compared to the controls (Table 2). If we assume a normal distribution, a single value may be considered as an outlier if it falls outside the two or three times the standard deviation. Outside the 2 × SD of RC patients’ Gaussian distribution were exclusively cancer patients. 6% of the RC patients have increased values in preexisting and remaining γH2AX foci and 6% have exclusively increased remaining γH2AX foci. 5.8% of the RC patients and 1.7% of the healthy individuals had solely increased preexisting γH2AX foci (Figure 3D).

**Table 2:** **Percentage of healthy individuals and patients outside of one to three standard deviations from the mean of a Gaussian distribution**

**Group**	**Healthy individuals**		**Rectal cancer patients**		**Rectal cancer patients**	
**Distribution**	**Healthy individuals**		**Rectal cancer patients**		**Healthy individuals**	
**Exposure**	**Preexisting**	**2 Gy, 24 h**	**Preexisting**	**2 Gy, 24 h**	**Preexisting**	**2 Gy, 24 h**
mean	0.49	2.63	0.59	2.97	-	-
>1× SD	17.2%	11.9%	21.9%	21.2%	42.3%	33.6%
>2× SD	5.2%	3.4%	14.6%	8.8%	22.6%	14.6%
>3× SD	3.4%	0.0%	5.1%	2.9%	18.2%	8.8%

Group defines the used healthy individuals or patients group. Distribution defines the used healthy individual’s distribution or the rectal cancer patient’s distribution to calculate the percentage of patients outside the one to three standard deviations. Mean is the mean value of the standard deviation.

## Discussion

In this study we compared the preexisting, induced and residual DNA double strand breaks in lymphocytes derived from 136 RC patients and 59 healthy individuals. We found distinct increased values of preexisting and remaining γH2AX foci in the group of RC patients. There are clear interindividual differences in both groups and we did find outliers in about 12% of the RC patients after ex vivo IR. 6% of the RC patients showed increased numbers of preexisting γH2AX foci. In the healthy individuals group there was only one individual (1.7%) with increased preexisting numbers of γH2AX foci. We could not correlate the measured values with radiotherapy related late effects because of the short period since IR exposure of patients. It is therefore difficult to judge whether the outliers have an increased radiosensitivity and an increased risk of therapy related late effects. Nevertheless, there is an unambiguous difference between the patients group having a distinct increased number of outliers compared to the healthy individuals. We found three different outlier groups (Figure 3D). One group (n = 8) has solely increased preexisting γH2AX foci and no raised values after ex vivo irradiation. We suggest that in this group the values are increased due to an elevated exposure of DNA damaging agents and not to an impaired DNA damage response. The second group (n = 8) are individuals having increased preexisting γH2AX foci and likewise increased γH2AX foci after ex vivo irradiation. Here we would argue that patients have an impaired DNA damage response and therefore already raised preexisting γH2AX foci and as a consequence increased values after ex vivo irradiation. In the third group (n = 8) solely the residual DNA DSB values are raised. Here an impaired DNA damage response may be sufficient to repair the rarely appearing spontaneous DNA damages, however is not sufficient to repair the numerous DNA double strand breaks induced by a dose of 2 Gy. This indicates that the outliers identified by ex vivo irradiation may have an impaired DNA damage response and probably an increased risk to develop radiotherapy related side effects. It is postulated that differences in the repair of DNA DSBs could possibly be related to differences in radiation sensitivity among individuals [11].

Some studies reported an association between γH2AX foci and individual radiosensitivity. Fleckenstein et al. found a correlation between a serious mucositis and a rising number of foci 24 hours after ex vivo irradiation [12]. Similar Li et al. found that patients with severe oral mucositis had increased remaining damage 24 h after ex vivo irradiation compared to patients with mild oral mucositis [13]. Another study on a breast cancer patients group with an adverse acute skin reaction (grade 3) to radiotherapy showed significantly increased radiation-induced γH2AX foci [14]. Additionally the disappearance of the foci was delayed compared to the group of breast cancer patients with normal skin reaction (grade 0–1) [14]. Another study showed clear differences in DNA repair capacity reflected by γH2AX foci formation in cells from a high proportion of apparently normal individuals using a low dose-rate assay [15]. In addition the persistence of γH2AX foci after the induction of DNA damage suggests that some of the damage remains unrepaired, which makes γH2AX an attractive candidate for the rapid assessment of radiation sensitivity in individuals and cell lines [16]. This may lead to the identification of cell lines and human subjects with defective DNA repair [17,18].

On the other hand there have been some studies reporting contradictory results and showing some weakness in the γH2AX assay. One group stated that γH2AX focus measurement has limited scope as a pre-RT predictive assay in lymphoblast cell lines from RT patients; however, the assay can successfully identify DNA DSB repair-defective patient’s cell lines [19]. Werbrouck et al. concluded from a gynaecological cancer collective that scoring γH2AX foci in isolated T lymphocytes after ex vivo irradiation is not predictive for late radiotoxicity [20]. The same authors confirmed in another study that no correlation was found between the γH2AX foci kinetics and the risk for acute normal tissue complications among patients during IMRT treatment for head and neck cancer [21]. With these contradictory results, Ivashkevich et al. suggested further validation of the assay to show whether the method is specific enough to be predictive in the identification of radiosensitive patients. They also supported the idea that even if the correlation between the assay and clinical radiosensitivity is incomplete, the ability of the assay to detect that subset of radiosensitive patients with defective DNA DSB repair pathways would be valuable per se [22].

There is much hope that the γH2AX assay has the capability to predict individual radiosensitivity [11,22]. The analysis of chromosomal aberrations has been proved to predict individual radiosensitivity. It is another lymphocyte based assay [23,24] however it takes several days and is work-intensive. The γH2AX assay can be performed quickly and can also be readily automated [22]. Recently, the automatic analyzes of γH2AX foci was reported [25,26]. Our approach of processing samples is fairly similar, yet with a lower level of automation. The main difference may be that in our system the software marks cells and foci and the user must finally accept or reject these selections. Technical problems with counting foci in the rounded lymphocytes we have overcome by using focal plane merging [27]. We used composite images with an extended depth of field consisting of five optical planes.

## Conclusions

In conclusion the γH2AX assay has the capability to identify a group of outliers which are probably patients with increased radiosensitivity which have the highest risk suffering from radiotherapy-related late sequelae. Future follow-up on these patients will correlate ex vivo data with clinical outcome.
